# Ambulatory Blood Pressure Monitoring in Children: A Cross-Sectional Study of Blood Pressure Indices

**DOI:** 10.3390/children12070939

**Published:** 2025-07-16

**Authors:** Sulaiman K. Abdullah, Ibrahim A. Sandokji, Aisha K. Al-Ansari, Hadeel A. Alsubhi, Abdulaziz Bahassan, Esraa Nawawi, Fawziah H. Alqahtani, Marwan N. Flimban, Mohamed A. Shalaby, Jameela A. Kari

**Affiliations:** 1 Pediatric Department, College of Medicine, Taibah University, Madinah 41541, Saudi Arabia; tu4101747@taibahu.edu.sa (S.K.A.); isandokji@gmail.com (I.A.S.); 2 Pediatric Nephrology Center of Excellence, Pediatric Nephrology Unit, Pediatric Department, King Abdulaziz University, Jeddah 21589, Saudi Arabia; aikalansari@moh.gov.sa (A.K.A.-A.); hadeelaalsubhi@gmail.com (H.A.A.); ambahassan@kau.edu.sa (A.B.); marwanfilemban@gmail.com (M.N.F.); mashalabi@kau.edu.sa (M.A.S.); 3 Pediatric Department, King Abdulaziz University Hospital, Jeddah 21589, Saudi Arabia; enawawi@kau.edu.sa; 4 Khamis Mushait Maternity & Children Hospital, Khamis Mushait 62454, Saudi Arabia; falqahatani114@moh.gov.sa

**Keywords:** pediatric hypertension, ambulatory blood pressure monitoring, cardiovascular risk, arterial stiffness, blood pressure variability, chronic kidney disease

## Abstract

**Background:** Ambulatory blood pressure monitoring (ABPM) is increasingly recognized as a more reliable indicator of blood pressure status in children than clinic-based measurements, with superior predictive value for cardiovascular morbidity and mortality. However, evidence on the clinical utility of ABPM-derived indices, such as pulse pressure (PP), pulse pressure index (PPI), rate pressure product (RPP), ambulatory arterial stiffness index (AASI), and average real variability (ARV), remains underexplored in the pediatric population, particularly among children with chronic kidney disease (CKD). **Objective:** To evaluate the correlation between ABPM-derived indices in children, with a subgroup analysis comparing those with and without CKD. Secondary objectives included identifying factors associated with AASI and ARV and assessing their utility in cardiovascular risk stratification. **Methods:** In this bicentric cross-sectional study, 70 children (41 with CKD and 29 controls) were enrolled. ABPM indices (PP, PPI, RPP, AASI, and ARV) were calculated, and both descriptive and inferential statistical analyses, including linear regression, were performed. **Results:** Systolic and diastolic hypertension were significant predictors of elevated ARV (*p* < 0.05), while body mass index (BMI) and glomerular filtration rate (GFR) were positively associated with AASI (*p* < 0.05). Use of angiotensin-converting enzyme inhibitors (ACEIs) was associated with reduced arterial stiffness (*p* = 0.02). Significant differences were observed in weight, BMI, PP, and PPI between the CKD and non-CKD groups, with ABPM demonstrating greater sensitivity in detecting vascular health markers. **Conclusions:** ABPM-derived indices, particularly PP, PPI, and ARV, show promise in improving cardiovascular risk assessment in children. These findings support the broader use of ABPM metrics for refined cardiovascular evaluation, especially in pediatric CKD.

## 1. Introduction

Hypertension is a well-established risk factor for cardiovascular and chronic kidney disease (CKD) [1]. Ambulatory blood pressure monitoring (ABPM) in children and adolescents is increasingly regarded as a more reliable indicator of blood pressure (BP) status than clinic-based BP measurements, due to its stronger correlation with target organ damage in adult and pediatric populations [2,3]. Furthermore, elevated BP during childhood has been shown to track into adulthood, increasing the risk of premature cardiovascular disease, left ventricular hypertrophy, and arterial stiffness later in life [3]. Recent studies suggest that 24 h ABPM is a better predictor of cardiovascular morbidity and mortality than office-based BP measurements [4]. Overweight children and adolescents may benefit from the broader adoption of ABPM. [5]. Routine integration of ABPM in the assessment of these children would enable the accurate identification of individuals with persistent ambulatory hypertension, masked hypertension, or reduced nocturnal dipping, facilitating early identification of those who may require prompt initiation of pharmacologic antihypertensive therapy [5]. This is especially relevant given that masked hypertension and abnormal dipping patterns are frequently missed in clinic-based assessments, yet are strongly associated with subclinical target organ damage in the pediatric population [4].

Several indices have been proposed to aid in the diagnosis and management of hypertension, including pulse pressure (PP), pulse pressure index (PPI), rate pressure product (RPP), ambulatory arterial stiffness index (AASI), and average real variability (ARV) [6,7,8]. Recent refinements in the definitions of ambulatory hypertension have incorporated predictive elements and raised the blood pressure threshold sensitivity, which facilitates more accurate diagnosis [9]. However, there is insufficient evidence evaluating the utility of all these indices in children, particularly those with CKD, who are known to exhibit increased arterial stiffness, impaired vascular compliance, and elevated cardiovascular risk even at a young age [10]. How these indices behave in pediatric CKD may help guide early interventions and improve long-term cardiovascular outcomes.

This study aims to evaluate the correlation between various ABPM indices in children, with a focus on their association with cardiovascular risk factors such as BMI, GFR, and hypertension status. A secondary aim is to compare these indices between children with and without CKD and explore predictors of AASI and ARV.

## 2. Materials and Methods

### 2.1. Study Design and Setting

This bicentric cross-sectional study was conducted at King Abdulaziz University Hospital in Jeddah and the Maternity and Children’s Hospital in Madinah. The study population included two pediatric groups: children withCKD and a control group with healthy kidney function. Groups were age- and gender-matched to reduce confounding and enhance comparability.

### 2.2. Participants and Data Collection

Children with CKD were identified through the pediatric nephrology clinic database, and controls were selected from those referred for routine evaluation without kidney disease. The Inclusion was based on the availability of ABPM data from 1 April 2023 to 1 February 2025. No formal sample size calculation was performed due to the exploratory nature of the study. All eligible patients within the study period were included.

Inclusion criteria were:

(1) Aged between 5 and 18 years;
(2) The presence ofCKD or elevated office blood pressure readings;
(3) Previously diagnosed hypertension based on office BP, or follow-up for hypertension risk;
(4) Availability of valid 24 h ABPM data.

Children under the age of 5 were not included, as ABPM is not applicable in this age group according to institutional protocol.

Exclusion criteria were:

(1) Children who were acutely hospitalized at the time of ABPM;
(2) Incomplete ABPM records with fewer than 75% valid readings;
(3) Missing clinical or laboratory data required for analysis.

Data collection included age at presentation, anthropometric measurements (weight, height, and body mass index), and a comprehensive set of laboratory tests, including initial and current serum creatinine levels and glomerular filtration rates (GFR). These measurements allowed for a thorough comparative analysis of kidney function among the cohorts. Incomplete ABPM readings and missing clinical data were excluded from the final analysis.

CKD was defined according to the K/DOQI workgroup guidelines, which specify that it is characterized by structural or functional abnormalities of the kidneys lasting for three months or more, or by a GFR of less than 60 mL/min per 1.73 m_2_ for the same duration, with or without additional signs of kidney damage [11].

The control group consisted of children without CKD who were referred for ABPM due to elevated office BP, family history of hypertension, or obesity. Some of these patients were on antihypertensive therapy based on prior diagnoses or ABPM-confirmed hypertension.

### 2.3. Blood Pressure Assessment and Definition of Hypertension

Hypertension definitions vary by age: in children under 13 years, it is defined as blood pressure exceeding the 95th percentile for age, gender, and height, or ≥120/80 mmHg, whichever is lower. In adolescents, adult criteria are applied (≥120/80 mmHg) [12]. Blood pressure measurements were taken during clinic visits, including systolic and diastolic pressures, pulse pressure, and pulse pressure index. A 24-h ambulatory blood pressure monitoring (ABPM) was also conducted using the Tonoport with Cardiosoft V7 software (version VI: GE Healthcare, Chicago, IL, USA), capturing various parameters such as blood pressure load and nocturnal dipping patterns. The study also assessed the prevalence of masked, white coat, and sustained ambulatory hypertension across the groups.

### 2.4. Outcomes and Definitions

The rate pressure product (RPP), which indicates myocardial oxygen demand and cardiac workload, was calculated using the heart rate and systolic blood pressure (SBP) data for each reading and then averaged [13]. The ambulatory arterial stiffness index (AASI) was calculated using the formula of 1 minus the diastolic blood pressure (DBP) regression slope on SBP values [14]. Systolic and diastolic variability were assessed through the average real variability (ARV), which was calculated using the following formula [15]:

(1) $$ARV=\frac{1}{n-1}\sum_{i-1}^{n-1}\left|BPi+1-BPi\right|$$

The pulse pressure index (PPI) and pulse pressure (PP) were calculated for each reading and averaged to obtain the mean PP and PPI over 24 h.

### 2.5. Statistical Analysis

Descriptive and inferential statistics were utilized to analyze the data. Continuous variables were assessed for normality using the Shapiro–Wilk test, which determined whether parametric (independent samples t-tests) or non-parametric (Mann–Whitney U) tests should be used. Categorical variables were compared using Fisher’s exact test. Linear regression was employed to analyze factors associated with continuous outcomes. Data visualization was conducted using Python (version 3.12), leveraging libraries such as Matplotlib (version 3.8.4), Seaborn (version 0.13.2), and Pandas (version 2.2.2) to create plots and graphs illustrating key findings. Statistical analyses were performed using SPSS Statistics for Windows, version 27. A *p*-value of less than 0.05 was considered statistically significant.

### 2.6. Ethical Considerations

This research received ethical approval from the Biomedical Ethics Unit at King Abdulaziz University, with the registration number HA-02-J-008. Although patient consent was waived for this retrospective study, ABPM was conducted as part of clinical care, and in some cases, parents declined ABPM testing, which limited inclusion.

## 3. Results

We enrolled 70 children: 41 diagnosed with CKD and 29 without CKD (Table 1). Among the children with CKD, 58.6% were boys, and the mean age at presentation was 11.24 ± 3.74 years. Among the non-CKD children, 51.7% were boys, and the mean age was 12.03 ± 3.03 years. Significant differences in weight and BMI were observed between groups, with the CKD group displaying lower median values (25.7 [IQR: 20.7–40.8] kg and 15.42 [IQR: 13.96–19.32] kg, respectively) compared to the non-CKD group (48.5 [IQR: 25.2–73.75] kg and 22.1 [IQR: 16.59–28.78] kg, respectively) (*p* = 0.004 and *p* < 0.001, respectively).

**Figure 1:**
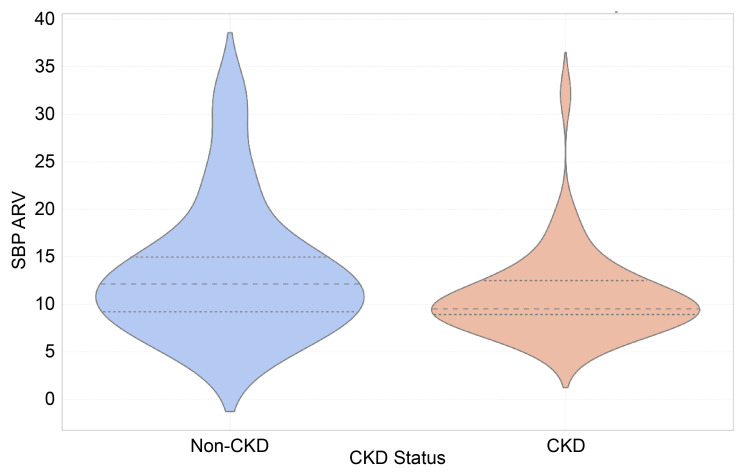
Violin plot of systolic blood pressure average real variability (SBP ARV) across CKD and non-CKD groups.

**Figure 2:**
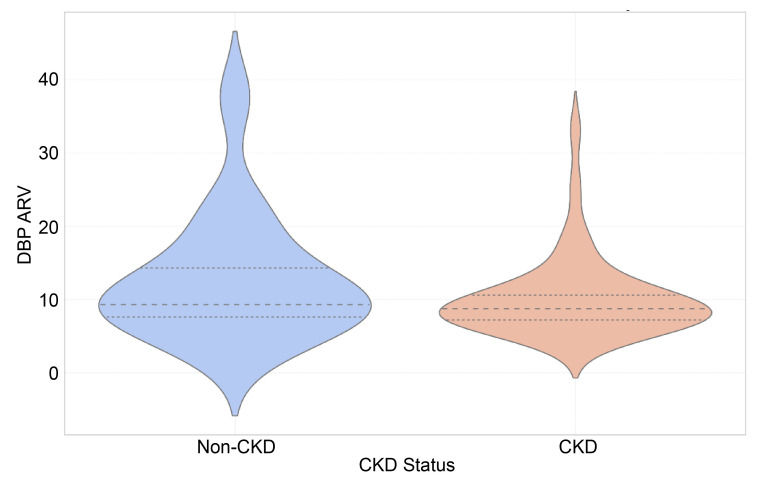
Violin plot of diastolic blood pressure average real variability (DBP ARV) across CKD and non-CKD groups.

The SBP and DBP ARV indices did not show significant differences between the groups, with SBP ARV medians of 9.52 [IQR: 8.86–12.52] for CKD and 12.12 [IQR: 8.74–15.29] for non-CKD (*p* = 0.08), as well as DBP ARV medians of 8.71 [IQR: 7.07–11.11] for CKD and 9.28 [IQR: 7.44–15.28] for non-CKD (*p* = 0.14) (Figure 1 and Figure 2).

**Figure 3:**
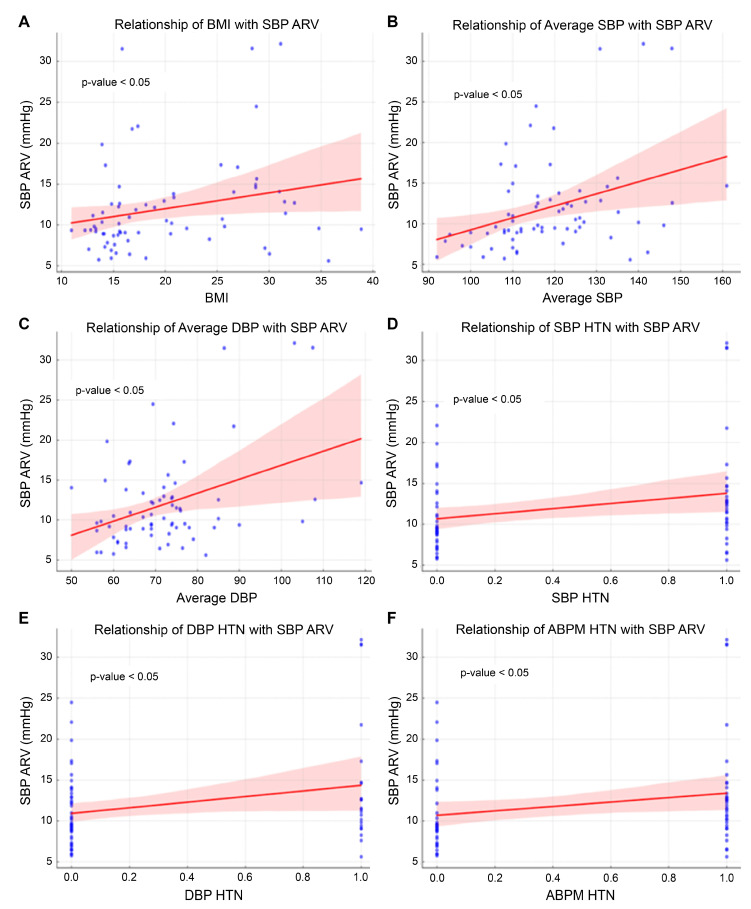
Linear regression of various factors on SBP ARV.

Linear regression analysis identified overall systolic and diastolic blood pressure as significant predictors of SBP ARV, with coefficients of 0.148 (95% CI: 0.054–0.241, *p* = 0.002) and 0.175 (95% CI: 0.079–0.271, *p* = 0.001), respectively (Figure 3). Additionally, the presence of systolic blood pressure hypertension (SBP HTN) and diastolic blood pressure hypertension (DBP HTN) was significantly associated with higher SBP ARV values, with coefficients of 3.112 (*p* = 0.024) and 3.432 (*p* = 0.019), respectively (Table 2).

**Figure 4:**
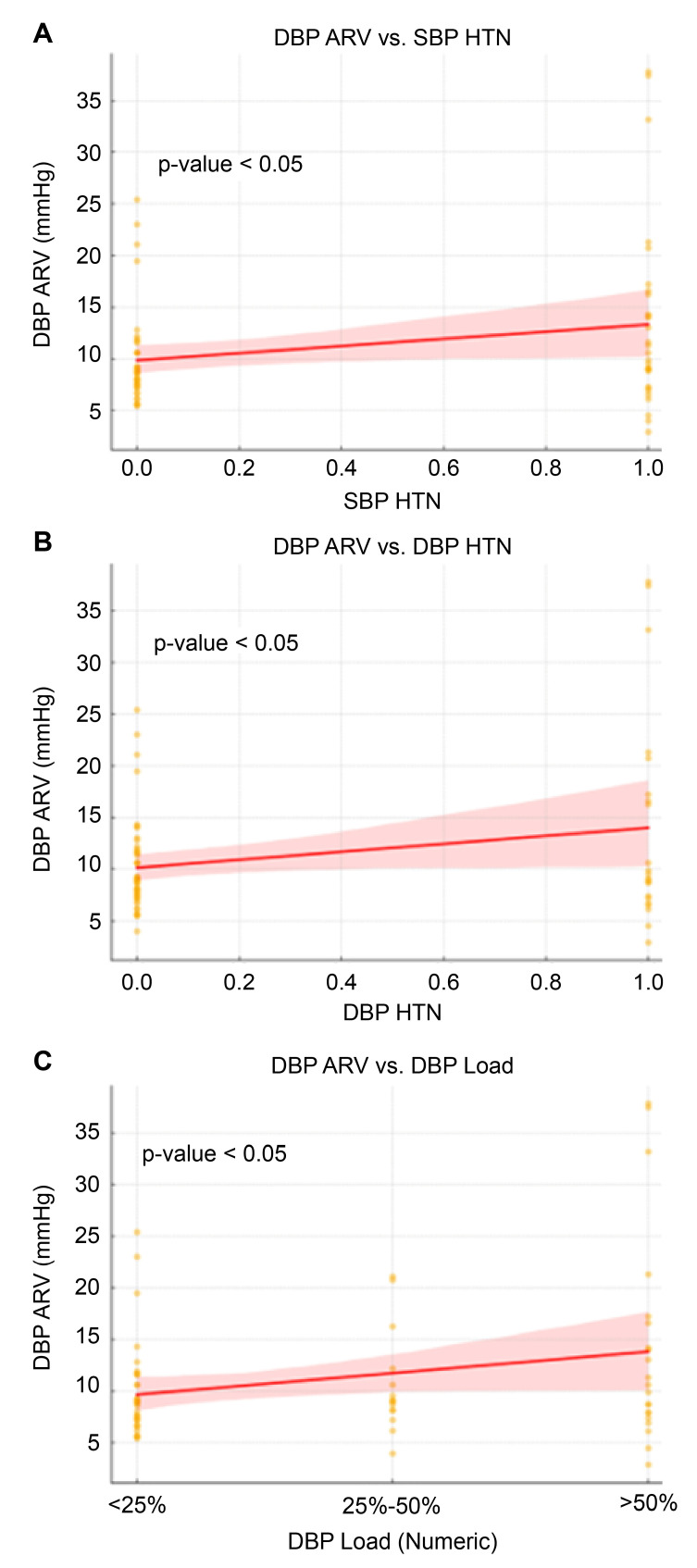
Linear regression of various factors on DBP ARV.

Linear regression analysis also identified SBP HTN and DBP HTN as significant predictors of DBP ARV among participants. Specifically, SBP HTN was associated with an increase in DBP ARV of 3.466 (95% CI: 0.164 to 6.768, p = 0.040), while DBP HTN showed an even stronger association, with an increase in DBP ARV of 3.856 (95% CI: 0.346 to 7.366, *p* = 0.032) (Figure 4, Table 3).

**Figure 5:**
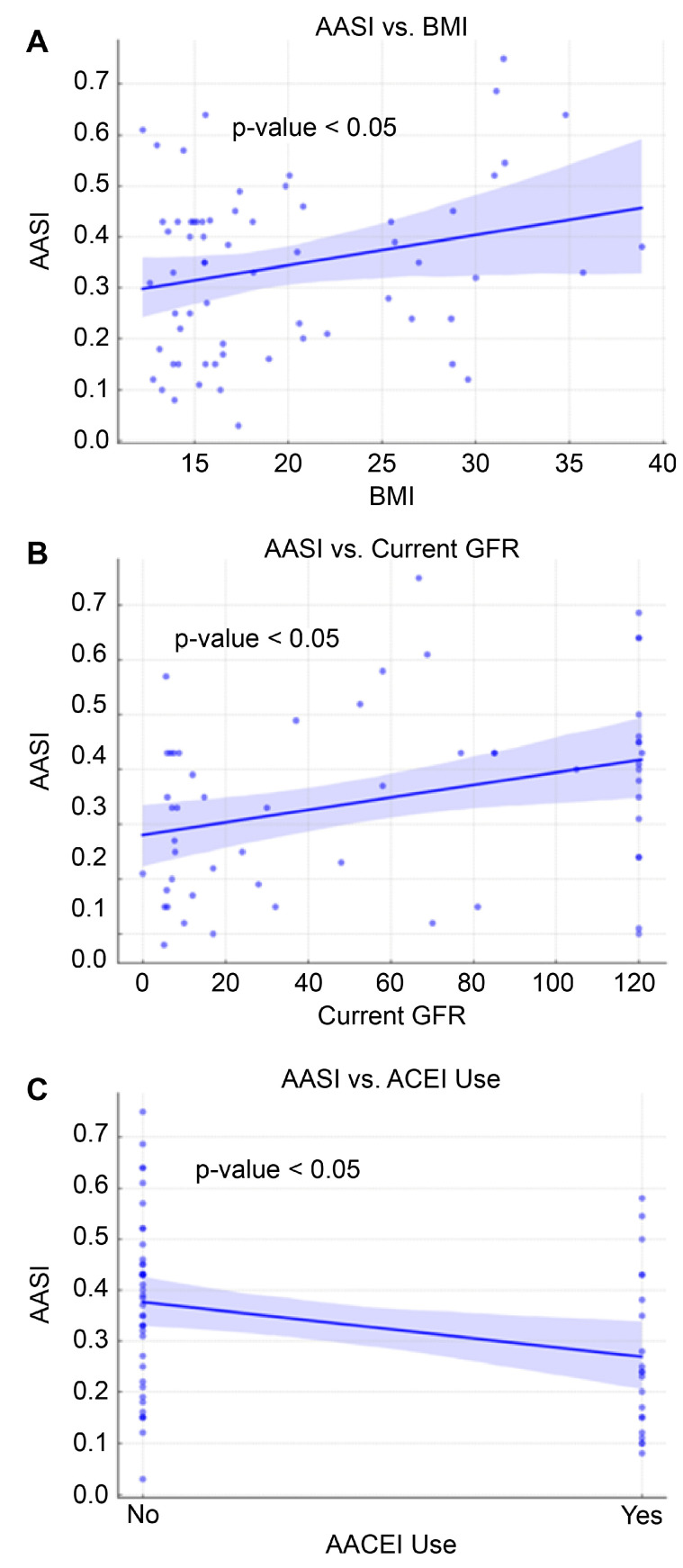
Linear regression of various factors on AASI.

The regression analysis of the AASI identified BMI and current GFR as significant predictors. Specifically, an increase in BMI was associated with an increase in AASI (B = 0.006, 95% CI: 0.001 to 0.012, *p* = 0.048), suggesting that higher BMI may contribute to increased arterial stiffness (Table 4 and Figure 5). Additionally, a positive association was found between current GFR and AASI (B = 0.001, 95% CI: 0.000 to 0.002, *p* = 0.006), indicating that better kidney function is associated with increased arterial stiffness levels.

The use of ACEIs was associated with lower AASI (B = −0.05, 95% CI: −0.09 to −0.01, *p* = 0.02). This finding supports the potential therapeutic benefits of ACEIs in reducing arterial stiffness (Figure 5). Other antihypertensive medications, including calcium channel blockers, beta-blockers, vasodilators, and centrally acting agents, did not significantly affect AASI. 

The use of antihypertensive medications was similar between the groups, with 58.9% of the CKD group and 44.8% of the non-CKD group using these medications (*p* = 0.33) (Table 1). Specific types of medications, such as ACEIs, ARBs, and CCBs, did not demonstrate significant differences in usage between the groups. 

Office PP and PPI values were significantly lower in the CKD group compared to the non-CKD group (45.6 ± 9.45 mmHg vs. 56.17 ± 14.24 mmHg, *p* = 0.002; and 0.36 ± 0.07 vs. 0.43 ± 0.08, *p* = 0.001, respectively). However, there were no significant differences in SBP and DBP measurements between the two groups in the office settings (*p* = 0.47 and *p* = 0.24, respectively) (Table 2 ABPM showed similar trends, with no significant differences in overall SBP and DBP, but a significant difference in ABPM PP (43.98 ± 8.33 mmHg in CKD vs. 50.53 ± 8.4 mmHg in non-CKD, *p* = 0.002).

## 4. Discussion

The findings of our bicentric cross-sectional study underscore the importance of ABPM in evaluating blood pressure indices among children, enhancing our understanding of its superiority over traditional office blood pressure measurements. This study aimed to systematically assess a wide range of ABPM-derived indices, with particular attention to their relationship with cardiovascular risk factors and kidney function. Our results highlight the complex interplay between obesity, hypertension, and vascular health in this pediatric population.

The SBP ARV was associated with BMI, DBP HTN, average SBP, SBP HTN, and ABPM HTN, while BMI and current GFR were directly associated with the AASI, and the use of ACEIs was inversely associated with AASI. DBP ARV was associated with SBP HTN, DBP HTN, and DBP load.

Our findings support the AASI as a key predictor of cardiovascular risk, aligning with evidence that higher BMI significantly contributes to arterial stiffness in children [16]. This is consistent with prior pediatric studies demonstrating that increased adiposity is linked to early vascular dysfunction [17]. The current study highlights factors influencing SBP and DBP variability. Notably, SBP HTN and DBP HTN significantly contribute to DBP variability, with DBP load solely increasing the DBP ARV, indicating a potential rise in cardiovascular risks for affected children [18]. Previous studies have also shown that BP variability, particularly elevated ARV, is an emerging marker of cardiovascular morbidity in both adult and pediatric populations [15,18]. Obesity increased the SBP ARV, which may indicate enhanced sympathetic nervous system activity [19]. These findings necessitate strict monitoring and control measures, emphasizing the need for targeted therapeutic interventions to mitigate these risks.

The observed association between higher GFR and increased AASI may reflect cross-sectional variability or early vascular adaptation in mild CKD, rather than actual improvement in kidney function over time. These dynamics emphasize the importance of a holistic approach to CKD management, which includes metabolic and hemodynamic interventions [10]. Importantly, this positive association may appear paradoxical, as better kidney function is typically associated with lower arterial stiffness. However, the finding may reflect residual confounding or early vascular changes that precede measurable renal impairment. Additionally, higher GFR in early CKD may represent a state of hyperfiltration, a compensatory but maladaptive process linked to vascular remodeling and increased stiffness [20]. These complexities underscore the limitations of cross-sectional interpretation and support the need for longitudinal studies to clarify the causal pathways. The differences in PP and PPI between the CKD and non-CKD groups highlight the potential of these metrics as indicators of vascular health and cardiovascular risk, which ABPM detects more reliably than office blood pressure (BP) measurements [21,22]. These findings support previous observations that children with CKD exhibit early vascular remodeling, even in the absence of overt clinical cardiovascular disease [10]. This insight is critical for providers caring for children with CKD.

Moreover, the significance of ABPM in identifying masked hypertension, white coat hypertension, and nocturnal dipping patterns cannot be overstated. These features provide valuable insights into daily patterns that may affect long-term cardiovascular health, suggesting a fundamental shift in the approach and management of pediatric hypertension [23]. Masked hypertension remains underrecognized in children but has been strongly linked to early target organ damage [4]. Looking forward, further research is necessary to explore the intersection of genetic, environmental, and clinical factors in managing hypertension for pediatric CKD patients. Research in personalized medicine could result in more effective, tailored therapies that consider individual differences in blood pressure responses and cardiovascular risk [24].

To the best of our knowledge, this is among the first studies in pediatrics to assess the full range of BP indices: RPP, AASI, PPI, PP, SBP ARV, and DBP ARV, while also considering other factors such as CKD, obesity, and antihypertensive medications.

This comprehensive approach adds to the limited existing literature on ABPM indices in children, particularly in those with CKD.

However, we encountered some limitations during this study, starting with the limited sample size due to the number of families who declined ABPM testing as part of clinical care, rather than due to research participation refusal and a lack of biological variables and cardiovascular status, as not all children underwent echocardiograms and electrocardiograms (ECGs). Additionally, the observational nature of this study limits its generalizability. Moreover, while we assessed arterial stiffness using validated surrogate indices such as the AASI, we were unable to perform direct measurements of arterial stiffness (e.g., pulse wave velocity or plethysmography) due to equipment limitations. We acknowledge that direct measurement would provide more robust data and consider this an important area for improvement in future research. Future research should involve a larger sample size and examine long-term outcomes, such as cardiovascular changes.

## 5. Conclusions

This study contributes novel data on the use of AASI and ARV in pediatric hypertension and CKD, offering a deeper understanding of early vascular changes beyond standard BP thresholds. Our findings suggest that specific ABPM-derived indices, such as AASI and ARV, may serve as useful adjuncts for assessing cardiovascular risk in children, particularly in those with CKD. These exploratory results warrant further investigation in larger, prospective cohorts.

## Figures and Tables

**Table 1:** Baseline characteristics of the study cohort.

	Total	CKD	Non-CKD	*p*-Value
Number (%)	70	41 (58.6)	29 (41.4)	
Gender (male, n [%])	38 (54.3)	23 (56.1)	15 (51.7)	0.81
Age (years, mean ± SD)	11.6 ± 3.5	11.2 ± 3.7	12.0 ± 3.0	0.34
Height (cm, mean ± SD)	136.5 ± 19.8	132.7 ± 18.6	141.9 ± 20.6	0.07
Weight kg (median [IQR])	30 [22–58.3]	25.7 [20.7–40.8]	48.5 [25.2–73.8]	0.004
BMI (median [IQR])	16.7 [14.7–25.9]	15.4 [14–19.3]	22.1 [16.6–28.8]	<0.001
Initial GFR (mL/min/1.73 m^2^ [IQR])	69 [23.36–120]	41.30 [12.87–95]	120 [84.75–120]	<0.001
Last GFR (mL/min/1.73 m^2^ [IQR])	50.3 [7.81–120]	17 [7.02–62.4]	120 [120–120]	<0.001
Hypertension studies				
Office SBP (mmHg, mean ± SD)	127.1 ± 16.4	124.8 ± 16.5	127.0 ±14.6	0.47
Office DBP (mmHg, median [IQR])	77 [66–86]	77 [67–88]	74 [65–83]	0.24
Office PPI (mean ± SD)	0.39 ± 0.08	0.36 ± 0.07	0.4 ± 0.1	0.001
Office PP (mean ± SD)	50.0 ± 12.7	45.6 ± 9.5	56.2 ± 14.2	0.002
ABPM Overall SBP (mmHg, mean ± SD)	118.6 ± 13.9	117.8 ± 15.1	119.8 ±12.2	0.99
ABPM Overall DBP (mmHg, median [IQR])	71 [63–76.1]	72 [63–79]	69 [61–74]	0.25
Office BP percentile (>95th, n [%])	33 (70.2)	25 (67.6)	8 (80)	0.70
ABPM PPI (median [IQR])	0.4 [0.36–0.45]	0.39 [0.3–0.42]	0.42 [0.39–0.45]	0.03
ABPM PP (mean ± SD)	46.7 ± 8.9	44.0 ± 8.3	50.5 ± 8.4	0.002
AASI (mean ± SD)	0.34 ± 0.17	0.34 ± 0.17	0.35 ± 0.16	0.37
RPP (median [IQR])	10,787 [9538–11,639]	10,650 [9265–11,562]	10,958 [9746–11,823]	0.60
SBP ARV (median [IQR])	10.2 [8.9–13.5]	9.5 [8.9–12.5]	12.1 [8.7–15.3]	0.08
DBP ARV (median [IQR])	9.0 [7.3–12.4]	8.71 [7.1–11.1]	9.3 [7.4–15.3]	0.14
SBP SD (median [IQR])	11.98 [10.19–15.8]	11.34 [10.17–14.85]	13.42 [10.68–19.12]	0.9
DBP SD (median [IQR])	10.95 [9.23–15.62]	10.75 [9.06–14.09]	11.45 [9.93–24.65]	0.19
ABPM Systolic percentile (>95th, n [%])	30 (42.9)	16 (39)	14 (48.3)	0.47
ABPM Diastolic percentile (>95th, n [%])	22 (31.4)	15 (36.6)	7 (24.1)	0.31
ABPM percentile (>95th, n [%])	34 (48.6)	20 (48.8)	14 (48.3)	>0.99
Masked Hypertension (n [%])	4 (5.7)	3 (7.3)	1 (3.4)	0.64
White Coat Hypertension (n [%])	19 (27.1)	10 (24.4)	9 (31)	0.59
Non-Dipping BP (Yes, n [%])	30 (42.9)	15 (36.6)	15 (51.7)	0.23
Antihypertensive medications				
Antihypertensive medications (Yes, n [%])	37 (52.9)	24 (58.9)	13(44.8)	0.33
ACEIs or ARBs (n [%])	23 (32.9)	13 (31.7)	10 (34.5)	>0.99
CCB (n [%])	21 (35.6)	15 (42.9)	6 (25)	0.18
BB (n [%])	6 (8.6)	5 (12.2)	1 (3.4)	0.40
Vasodilators (n [%])	5 (7.1)	4 (9.8)	1 (3.4)	0.40
Central acting (n [%])	1 (1.7)	1 (2.9)	0	>0.99
SBP Load				
<25% (n [%])	22 (31.4)	14 (34.1)	8 (27.6)	0.72
25–50% (n [%])	14 (20)	7 (17.1)	7 (24.1)	
>50% (n [%])	34 (48.6)	20 (48.8)	14(48.3)	
DBP Load				
<25% (n [%])	34 (48.6)	18 (43.9)	16 (55.2)	0.59
25–50% (n [%])	15 (21.4)	9 (22)	6 (20.7)	
>50% (n [%])	21 (30)	14 (34.1)	7 (24.1)	

Abbreviations: BMI, body mass index; SBP, systolic blood pressure; DBP, diastolic blood pressure; PPI, pulse pressure index; PP, pulse pressure; ABPM, ambulatory blood pressure monitoring; AASI, arterial stiffness index; RPP, rate pressure product; ARV, average real variability; SD, standard deviation; ACEI, angiotensin-converting enzyme inhibitor; CCB, calcium channel blocker; BB, beta-blocker.

**Table 2:** Linear regression of factors associated with SBP ARV.

Variable	OR	95% CI Lower	95% CI Upper	*p*-Value
Age	−0.09	−0.49	0.31	0.667
Weight	0.05	−0.01	0.10	0.132
Height	0.02	−0.05	0.09	0.592
BMI	0.19	0.00	0.39	0.049
Initial GFR	−0.01	−0.03	0.02	0.810
Current GFR	0.01	−0.01	0.04	0.190
Overall SBP	0.15	0.05	0.24	0.002
Overall DBP	0.18	0.08	0.27	0.001
SBP HTN	3.11	0.42	5.80	0.024
DBP HTN	3.43	0.57	6.29	0.019
ABPM HTN	2.71	0.02	5.39	0.049
Dipping	−1.40	−4.17	1.38	0.319
WCH	−0.60	−3.70	2.51	0.702
Masked	−0.03	−5.99	5.93	0.992
ASSI High	1.94	−2.62	6.51	0.398
AASI	2.45	−5.72	10.62	0.552
SBP Load	1.03	−0.53	2.58	0.19
DBP Load	1.55	−0.01	3.10	0.051
PPI	−4.09	−12.62	4.44	0.34
PP	5.07	−0.16	0.16	0.99
Office SBP	0.03	−0.06	0.11	0.514
Office DBP	0.03	−0.07	0.13	0.545
Non-CKD	2.62	−0.12	5.35	0.06
CCB	−0.47	−1.24	0.3	0.23
ACEI	0.28	0.71	−1.19	1.75
BB	0.55	−0.43	1.53	0.27
Vasodilators	0.87	0.00	1.74	0.05
Central acting	0.08	−1.58	1.75	0.92

Abbreviations: BMI, body mass index; GFR, glomerular filtration rate; SBP, systolic blood pressure; DBP, diastolic blood pressure; PPI, pulse pressure index; PP, pulse pressure; ABPM, ambulatory blood pressure monitoring; AASI, arterial stiffness index; ARV, average real variability; ACEI, angiotensin-converting enzyme inhibitor; CCB, calcium channel blocker; BB, beta-blocker.

**Table 3:** Linear regression of factors associated with DBP ARV.

Variable	OR	95% CI Lower	95% CI Upper	*p*-Value
Age	−0.26	−0.75	0.23	0.29
Weight	0.04	−0.04	0.11	0.31
Height	0.01	−0.08	0.09	0.858
BMI	0.18	−0.06	0.41	0.14
Initial GFR	−0.02	−0.03	0.03	0.87
Current GFR	0.02	−0.01	0.04	0.25
SBP HTN	3.47	0.16	6.77	0.04
DBP HTN	3.86	0.35	7.37	0.03
ABPM HTN	2.61	−0.70	5.93	0.12
Dipping	−1.44	−4.83	1.95	0.40
WCH	−1.76	−5.53	2.00	0.35
Masked	0.19	−7.08	7.45	0.96
ASSI High	2.67	−2.57	7.91	0.31
AASI	2.12	−7.35	11.58	0.66
SBP Load	1.12	−0.79	3.02	0.25
DBP Load	2.08	0.20	3.96	0.03
PPI	−6.16	−16.5	4.12	0.24
PP	−0.02	−0.21	0.17	0.80
Office SBP	0.01	−0.09	0.11	0.84
Office DBP	0.02	−0.11	0.14	0.77
Non-CKD	2.69	−0.67	6.05	0.11
CCB	−0.33	−1.28	0.61	0.48
ACEI	0.39	−1.4	2.19	0.66
BB	0.98	−0.20	2.16	0.10
Vasodilators	0.98	−0.09	2.04	0.07
Central acting	−0.57	−2.60	1.45	0.58

BMI, body mass index; SBP, systolic blood pressure; DBP, diastolic blood pressure; PPI, pulse pressure index; PP, pulse pressure; ABPM, ambulatory blood pressure monitoring; AASI, arterial stiffness index; ARV, average real variability; ACEI, angiotensin-converting enzyme inhibitor; CCB, calcium channel blocker; BB, beta-blocker.

**Table 4:** Linear regression of factors associated with AASI.

Variable	OR	95% CI Lower	95% CI Upper	*p*-Value
Age	0.001	−0.01	0.013	0.825
Weight	0.002	0.000	0.003	0.06
Height	0.001	−0.001	0.003	0.48
BMI	0.006	0.001	0.012	0.048
Initial GFR	0.001	0.000	0.002	0.16
Current GFR	0.001	0.000	0.002	0.006
SYS HTN	0.06	−0.02	0.14	0.15
DBP HTN	−0.002	−0.09	0.09	0.97
ABPM HTN	0.063	−0.02	0.15	0.13
Dipping	−0.03	−0.11	0.06	0.54
WCH	−0.09	−0.18	−0.004	0.04
Masked	−0.04	−0.22	0.13	0.62
SBP Load	0.01	−0.04	0.05	0.81
DBP Load	−0.01	−0.05	0.04	0.82
PPI	0.31	0.07	0.54	0.013
PP	0.004	−0.001	0.008	0.12
Office SBP	−0.001	−0.004	0.001	0.36
Office DBP	−0.002	−0.005	0.001	0.28
Non-CKD	−0.02	−0.101	0.07	0.72
CCB	−0.01	−0.033	0.012	0.34
ACEI	−0.05	−0.09	−0.01	0.02
BB	−0.02	−0.05	0.01	0.18
Vasodilators	−0.01	−0.04	0.02	0.35
Central acting	0.01	−0.04	0.06	0.60

BMI, body mass index; SBP, systolic blood pressure; DBP, diastolic blood pressure; PPI, pulse pressure index; PP, pulse pressure; ABPM, ambulatory blood pressure monitoring; AASI, arterial stiffness index; ACEI, angiotensin-converting enzyme inhibitor; CCB, calcium channel blocker; BB, beta-blocker; OR, odds ratio.

## Data Availability

The original contributions presented in this study are included in the article. Further inquiries can be directed to the corresponding author.

## Abbreviations

The following abbreviations are used in this manuscript:

ABPM	Ambulatory Blood Pressure Monitoring
AASI	Ambulatory Arterial Stiffness Index
ARV	Average Real Variability
BMI	Body Mass Index
BP	Blood Pressure
CKD	Chronic Kidney Disease
DBP	Diastolic Blood Pressure
GFR	Glomerular Filtration Rate
PP	Pulse Pressure
PPI	Pulse Pressure Index
RPP	Rate Pressure Product
SBP	Systolic Blood Pressure
ACEI	Angiotensin-Converting Enzyme Inhibitor
ARB	Angiotensin II Receptor Blocker
CCB	Calcium Channel Blocker
BB	Beta-Blocker
SD	Standard Deviation
WCH	White Coat Hypertension
